# Malaria Case-Management following Change of Policy to Universal Parasitological Diagnosis and Targeted Artemisinin-Based Combination Therapy in Kenya

**DOI:** 10.1371/journal.pone.0024781

**Published:** 2011-09-14

**Authors:** Andrew Nyandigisi, Dorothy Memusi, Agneta Mbithi, Newton Ang'wa, Mildred Shieshia, Alex Muturi, Raymond Sudoi, Sophie Githinji, Elizabeth Juma, Dejan Zurovac

**Affiliations:** 1 Division of Malaria Control, Ministry of Public Health & Sanitation, Nairobi, Kenya; 2 Rift Valley Provincial General Hospital, Ministry of Public Health & Sanitation, Nakuru, Kenya; 3 Management Sciences for Health, Nairobi, Kenya; 4 Malaria Public Health & Epidemiology Group, Centre for Geographic Medicine Research - Coast, Kenya Medical Research Institute/Wellcome Trust Research Programme, Nairobi, Kenya; 5 Centre for Tropical Medicine, Nuffield Department of Clinical Medicine, University of Oxford, Oxford, United Kingdom; 6 Center for Global Health and Development, Boston University School of Public Health, Boston, Massachusetts, United States of America; State University of Campinas, Brazil

## Abstract

**Background:**

The change of malaria case-management policy in Kenya to recommend universal parasitological diagnosis and targeted treatment with artemether-lumefantrine (AL) is supported with activities aiming by 2013 at universal coverage and adherence to the recommendations. We evaluated changes in health systems and case-management indicators between the baseline survey undertaken before implementation of the policy and the follow-up survey following the first year of the implementation activities.

**Methods/Findings:**

National, cross-sectional surveys using quality-of-care methods were undertaken at public facilities. Baseline and follow-up surveys respectively included 174 and 176 facilities, 224 and 237 health workers, and 2,405 and 1,456 febrile patients. Health systems indicators showed variable changes between surveys: AL stock-out (27% to 21%; p = 0.152); availability of diagnostics (55% to 58%; p = 0.600); training on the new policy (0 to 22%; p = 0.001); exposure to supervision (18% to 13%; p = 0.156) and access to guidelines (0 to 6%; p = 0.001). At all facilities, there was an increase among patients tested for malaria (24% vs 31%; p = 0.090) and those who were both tested and treated according to test result (16% to 22%; p = 0.048). At facilities with AL and malaria diagnostics, testing increased from 43% to 50% (p = 0.196) while patients who were both, tested and treated according to test result, increased from 28% to 36% (p = 0.114). Treatment adherence improved for test positive patients from 83% to 90% (p = 0.150) and for test negative patients from 47% to 56% (p = 0.227). No association was found between testing and exposure to training, supervision and guidelines, however, testing was significantly associated with facility ownership, type of testing, and patients' caseload, age and clinical presentation.

**Conclusions:**

Most of the case-management indicators have shown some improvement trends; however differences were smaller than expected, rarely statistically significant and still leaving a substantial gap towards optimistic targets. The quantitative and qualitative improvement of interventions will ultimately determine the success of the new policy.

## Introduction

Universal parasitological testing and subsequent treatment of test positive patients with artemisinin-based combination therapy (ACT) are the critical components of the latest international recommendations for malaria case-management [1]. However, the success of the implementation of the new case-management policy is dependent upon series of factors of which availability of commodities at health facilities and case-management practices are of vital importance to ensure cost-benefit of the diagnostics and ACT based case-management strategies [2]–[4].

In 2009, Kenya launched the new 2009–2017 National Malaria Strategy (NMS) whose case-management mainstay is parasitological testing of all febrile patients across all age groups and areas of malaria endemicity and treatment of only test positive patients with nationally recommended ACT – artemether-lumefantrine (AL) [5], [6]. Simultaneously, by 2013, the new NMS specified programmatic directions to ensure universal availability of AL and malaria diagnostics as well as universal health worker's adherence to the new malaria case-management guidelines [7]. In this manuscript we report levels and changes in the availability of commodities and malaria case-management practices between two national health facility surveys; the baseline survey undertaken at the beginning of 2010, prior to the implementation of the new NMS, and the follow-up survey undertaken at the end of 2010, following the first year of the implementation activities.

## Methods

### Description of the key 2010 implementation activities

The main implementation activity during 2010 was a nationwide training for front-line health workers on the new case-management policy. The training took place between April and September 2010. The training was implemented following the training-of-trainers two-stage cascade format, starting at the national level by training representatives of 10 organizations who, in 110 training sessions, trained 4,807 health workers in the public sector nationwide. The training was done outside of health facilities, in the form of 3-day workshops according to standardized training curriculum [8]. One day was devoted to the management of uncomplicated malaria. The teaching modalities included lectures and theoretical case scenarios. The training was based on the recommendations in the new guidelines for health workers which were disseminated to health workers after the training following the launch of the guidelines in September 2010. During the first year of the implementation, the activities related to the strengthening of malaria component of the routine supervisory activities were initiated through the finalization of the supervisory manuals and its limited implementation emphasizing supportive supervision of health workers on malaria case-management including observations of outpatient consultations. With respect to malaria diagnostics, distribution of rapid diagnostic tests (RDT) initiated in 2006 continued in 33 out of 149 districts, as well as on smaller scale through the non-governmental and faith-based organizations across the country. During the same period, malaria microscopy, the traditional diagnostic mainstay in Kenya, was on smaller scale supported across the country through the in-service training of malaria microscopists and strengthening of the quality assurance procedures. Finally, with the respect to the supply of ACTs, the distribution of artemether-lumefantrine (AL), the recommended first line treatment for management of uncomplicated malaria deployed in 2006, continued during 2010 through the routine government supply chains.

### Indicators

The rationale for the selection of key indicators was based on those ones specified in the new national Malaria Monitoring and Evaluation Plan 2009–2017 [7], those representing main deficiencies detected in past which can severely compromise the success of the new malaria case-management policy in Kenya [9], [10], and those ones that are relatively simple to collect over short period of time. The key indicators at health facility level included availability of AL, other antimalarials, malaria diagnostic services, national guidelines and basic equipment important for malaria case-management. The key indicators at health worker level were the proportions of health workers who received training on the new case-management recommendations and supervisory visit including any malaria case-management activity.

The primary study indicator was measured at the patient level and referred to the recommended testing and treatment management of uncomplicated malaria in line with the new national malaria guidelines for health workers. The new guidelines state that 1) “*all patients with fever or history of fever should be tested for malaria and only patients who test positive should be treated for malaria*” and that 2) “*the recommended first line treatment for uncomplicated malaria in Kenya is artemether-lumefantrine*” [6]. To reflect criteria for testing and AL treatment, we included febrile, non-pregnant patients weighing 5 kg and above, presenting for an initial outpatient visit without being referred or admitted for hospitalization. Guidelines do not specify recommended management of patients with test negative results. Therefore, our primary indicator of correct management was a composite performance from the malaria perspective that included all of the following criteria: 1) patient was tested for malaria; 2) if positive test result patient was treated with AL, and 3) if negative test result patient was not treated for malaria. The secondary outcomes reflected individual components of the case-management in various patients' subgroups including testing and treatment based on the use and result of malaria testing.

### Study design, sample size and sampling

The study design included two national, cross-sectional, cluster sample health facility surveys. The sample size was calculated to detect 15% change in the performance of the primary case-management indicator between two survey rounds. The sample size was adjusted to take into consideration clustering effect at the health facility level and the likelihood of practices at facilities with unavailable case-management commodities. Therefore, in order to detect 15% difference (from conservative estimates of 50% to 65%) with the level of confidence of 5%, power of 80%, design effect of 2, and assumption that 50% of facilities will not have either AL or malaria diagnostic services, the estimated sample size was 680 patients below and above 5 years of age during the each survey. Assuming that on average a minimum of 4 eligible patients will be recruited in each age group at each facility over one survey day, the minimum required number of surveyed facilities was 170 (680/4).

**Figure 1:**
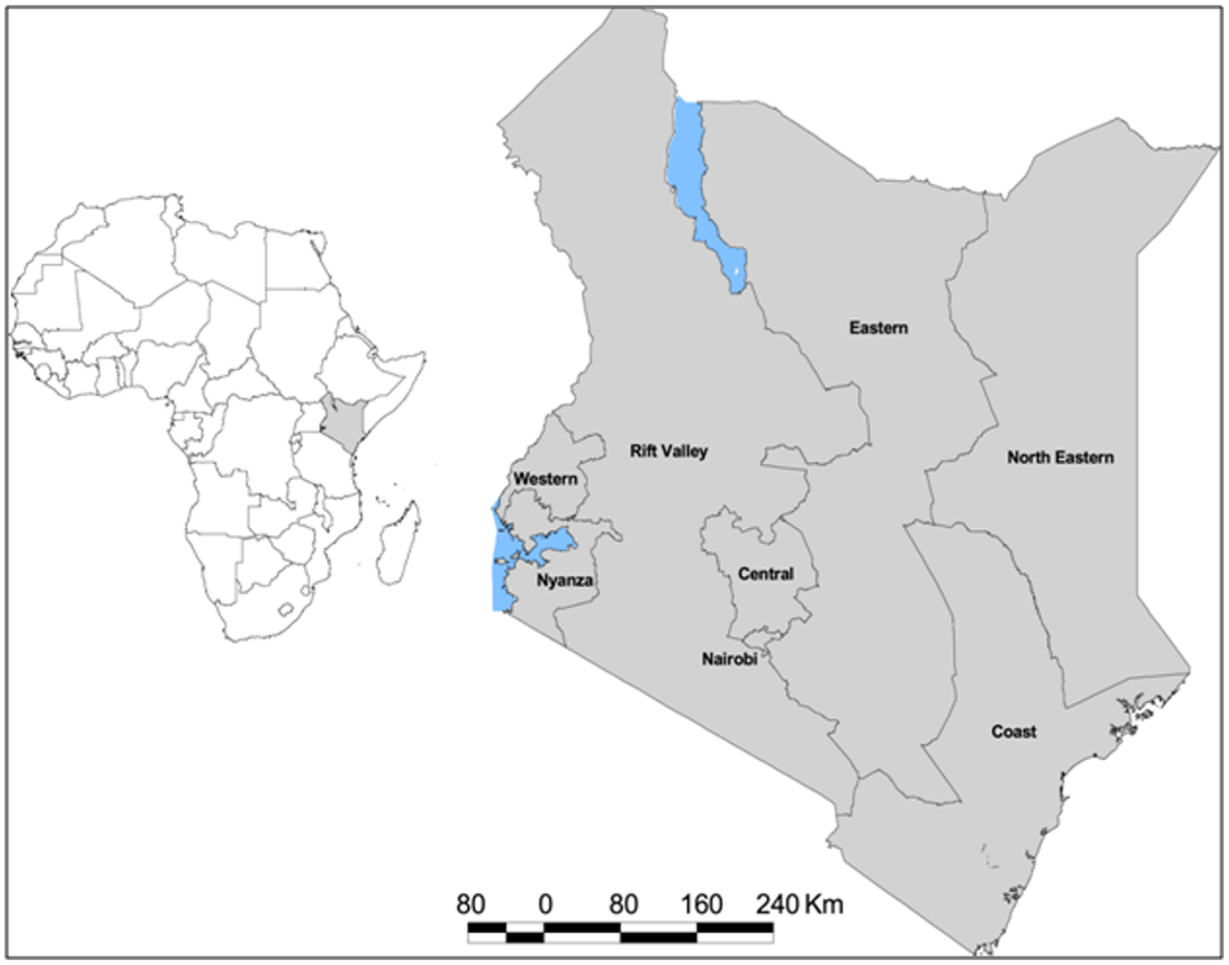
The map of Kenya showing provincial administrative boundaries and position within Africa.

During each survey, a national representativeness was assured drawing a stratified random sample from the universe of public facilities and taking into consideration administrative boundaries, type of facilities and their ownership. The following facilities were excluded from the survey: 1) facilities from Nairobi province requiring special studies to evaluate malaria case-management, 2) tertiary hospitals because they serve mainly as referral facilities, and 3) government facilities providing services to special patient groups such as military or prisoners. In each of seven provinces (Figure 1), four strata based on the facility type (hospitals versus smaller facilities) and ownership (government versus faith based/non-government) were formed. Finally, from each of the 28 strata, a simple, random sample proportional to the number of facilities in a stratum was drawn. A cluster was defined as all encounters between health workers and outpatients occurring on a single survey day.

### Data collection

The health facility surveys were conducted with ten teams each composed of three data collectors. In each team one surveyor was a team leader and performed facility assessment and interviews with health workers. The other two team members were student nurses who carried out exit interviews with outpatients. The training of data collectors and concordance testing was undertaken over five days. On the last day of the training, a field trial of was conducted at health facilities not included in the survey.

At each of the survey facilities data were collected over one survey day. Data were collected using three methods. First, all outpatients underwent rapid screening when they were leaving the facility. Upon obtaining written informed consent, non-referred and non-pregnant febrile patients presenting for an initial visit and weighing 5 kg and above proceeded with the interview during which information was collected from patient cards about malaria diagnostics requested, results reported and medications prescribed. During the interviews information was also collected about patients' age, weight, sex, temperature, duration of fever, main complaints, prior use of antimalarial drugs, and drug dispensing tasks performed. Second, at each facility the availability of antimalarial drugs, RDTs, functional malaria microscopy service, weighing scales and job-aides, was assessed. Finally, at the end of the working day all health workers who saw recruited patients on the survey day were interviewed to collect information on their demographics, pre-service training, and retrospective exposure to in-service training and supervision. Informed written consent was obtained for all health workers.

### Data management and analysis

Data entry and cleaning was undertaken using Access (Microsoft, USA). All forms were entered twice by independent data entry clerks. The analysis was performed using STATA, version 11 (StataCorp, College Station, Texas). Level estimates are presented for each survey as proportions, with corresponding 95% confidence intervals (CIs) adjusted for clustering at the health facility level. Changes in proportions between baseline and follow-up survey were tested using cluster adjusted chi-square test. Hypothesis testing and CI estimations were done with an alpha level of 0.05.

Descriptive analysis was undertaken at the health facility, health worker, and patient level. First, to assess coverage and exposure to interventions analysis was undertaken at health facility and health worker level. Second, to assess the overall performance of the new case-management policy practices were analyzed at patient level at all health facilities regardless of the availability of the case-management commodities. Third, to assess health workers adherence to the guidelines the same analysis was restricted to the facilities where AL and diagnostics were in stock on the day of the survey. Fourth, since the new case-management policy does not differ between age groups, the results are reported across all age groups while age specific results are available upon request to the authors.

Finally, to explore factors influencing low composite health workers' adherence (36%) following delivery of the interventions, the predictors analysis examining association between malaria testing and health facility, health worker and patient level factors was also performed at facilities with available commodities during the follow-up survey. Malaria testing outcome was specifically selected since low performance of this task provided an overwhelming contribution (78%) to non-adherent practices of the composite performance indicator. The logistic regression using generalized estimating equations with an independent working correlation matrix was applied to account for the correlated nature of the data. In the univariate analysis we first estimated odds ratios (OR), *P*-values and 95% CIs for the association between health workers' decision to test for malaria and the following factors: health facility type, ownership and type of available malaria testing; health workers' pre-service training; in-service training on the new case-management policy; access to national malaria guidelines; exposure to malaria supervision; patients' caseload on survey day; and patients' age, temperature and main complaints. Factors with *P*-value<0.15 were entered into multivariate model.

### Ethics statement

Ethical approval for the study was provided by the Kenyatta National Hospital/University of Nairobi-Ethics and Research Committee (reference number KNH-ERC/A/383). Informed written consent was obtained for all participants.

## Results

### Sample description

The baseline and follow-up surveys were respectively undertaken between January 18–February 12, 2010 and November 8–December 3, 2010. The baseline survey included 174 health facilities, 224 health workers, 2,405 patients who met inclusion criteria at all health facilities and 1,239 patients at facilities where AL and malaria diagnostic services were available. During the follow-up survey, 176 facilities were assessed, 237 health workers interviewed, and respectively 1,456 and 861 patients' consultations meeting the same criteria were evaluated at all facilities and facilities with AL and diagnostics in stock. During both surveys, the majority of assessed facilities were dispensaries (70.1% vs 66.5%), followed by health centres (18.4% vs 21.6%) and hospitals (11.5% vs 11.9%). Similarly, during both surveys the majority of facilities were government owned (73.0% vs 78.4%), followed by faith-based (25.9% vs 19.3%) and non-governmental organizations (1.2% vs 2.3%). With respect to health workers, the majority during both survey rounds were female (52.7% vs 53.2%) and nurses by cadre (63.0% vs 64.1%). Finally, the characteristics of recruited febrile patients were similar during both survey rounds. Most were 5 years and older (55.5% vs 53.6%), female (56.1% vs 53.8%), reporting to the health facility 3 days or more after the onset of illness (77.4% vs 74.0%) and without prior use of any antimalarial treatment for the current illness (95.0% vs 95.4%). During both surveys less than 1% of patients had completed AL dose before reporting to the facility. No health worker, adult patient or caretaker on behalf of sick child refused to participate in the study.

### Health facility and health worker readiness to implement new case-management policy

Table 1 presents survey levels and changes in the health facility and health worker readiness to implement new case-management policy. During both surveys functional weighing scales and thermometers were present at nearly all health facilities. Just above half of the facilities had capacity to provide parasitological malaria diagnosis, mainly relying on malaria microscopy. There were no significant changes in overall diagnostic capacities between surveys (55.2% vs 58.0%; p = 0.600), neither in the provision of malaria microscopy (50.6% vs 53.4%; p = 0.596) nor in the availability of RDTs (7.5% vs 8.5%; p = 0.717). Among facilities in the districts receiving RDTs since 2007, 35.7% (10/28) of facilities stocked RDTs during the baseline and 19.3% (6/31) during the follow-up survey.

**Table 1:** Levels and changes in health facility and health worker readiness to implement new case-management policy.

	Baseline survey		Follow-up survey		*P*-value
	n (%)	95% CI	n (%)	95% CI	
Health facility characteristics	N = 174		N = 176		
Availability of weighing scales	174 (100)	NA	174 (98.9)	97.3–100	0.158
Availability of functional thermometer	158 (90.8)	86.5–95.1	159 (90.3)	86.0–94.7	0.882
Availability of new national guidelines	0	NA	10 (5.7)	2.2–9.1	0.001
Availability of malaria diagnostics					
Functional microscopy	88 (50.6)	43.1–58.1	94 (53.4)	46.0–60.9	0.596
Non-expired malaria RDT	13 (7.5)	3.5–11.4	15 (8.5)	4.4–12.7	0.717
Any functional diagnostics	96 (55.2)	47.7–62.6	102 (58.0)	50.6–65.3	0.600
Availability of AL on survey day					
At least one AL pack	164 (94.3)	90.8–97.7	171 (97.2)	94.7–99.6	0.180
AL 6 pack	141 (81.0)	75.2–86.9	157 (89.2)	84.6–93.8	0.032
AL 12 pack	139 (79.9)	73.9–85.9	152 (86.4)	81.2–91.5	0.106
AL 18 pack	138 (79.3)	73.2–85.4	144 (81.8)	76.1–87.6	0.553
AL 24 pack	150 (86.2)	81.0–91.4	153 (86.9)	81.9–92.0	0.842
All four AL packs	113 (64.9)	57.8–72.1	126 (71.6)	64.9–78.3	0.181
SP tablets	154 (88.5)	83.7–93.3	154(88.0)^a^	83.1–92.9	0.883
Quinine tablets	120 (69.0)	62.0–75.9	148(84.6)^a^	79.2–90.0	0.001
Quinine injections	135 (77.6)	71.3–83.8	147(84.5)^b^	79.0–90.0	0.160
AL stock-out in 3 months prior to the survey^a^					
All four AL packs	47 (27.2)^a^	20.5–33.9	36 (20.6)^a^	14.5–26.6	0.152
AL 6 pack	65 (37.6)^a^	30.3–44.9	53 (30.1)	23.3–37.0	0.141
AL 12 pack	76 (43.9)^a^	36.5–51.4	57 (32.4)	25.4–39.4	0.026
AL 18 pack	90 (52.0)^a^	44.5–59.5	74 (42.1)	34.7–49.4	0.062
AL 24 pack	68 (39.3)^a^	32.0–46.6	62 (35.2)	28.1–42.4	0.431
At least one AL pack	103 (59.5)^a^	52.2–66.9	92 (52.3)	44.8–59.7	0.192

Health worker characteristics	N = 224		N = 237		
Trained on new case-management policy	0	NA	51 (21.5)	16.2–26.8	<0.001
Exposure to supervisory visit (past 3 months)					
Had any type of visit	93 (41.5)	35.0–48.0	123 (51.9)	45.5–58.3	0.026
Had any visit including malaria CM	40 (17.9)	12.8–22.9	31 (13.1)	8.8–17.4	0.156
Had any visit including observations	15 (6.7)	3.4–10.0	16 (6.8)	3.5–10.0	0.981

RDT = rapid diagnostic test; AL = artemether-lumefantrine; CM = case management; NA = not applicable.

a Denominator does not include 1 health facility with missing value.

b Denominator does not include 2 health facilities with missing values.

During the baseline survey at least one AL pack was in stock at 94.3% of facilities while the availability of weight-specific AL packs ranged from 79.3% for 18 tablets pack to 86.2% for 24 tablets pack. All four AL packs were in stock at 64.9% of facilities. Yet, an increase trend, albeit statistically significant only for 6 tablets pack (81.0% vs 89.2%; p = 0.032), was observed between two surveys. With respect to AL stock-out in 3 months prior to the surveys, stock-out of all four AL tablet packs decreased from 27.2% to 20.6% (p = 0.152) and stock out of at least one AL pack decreased from 59.5% to 52.3% (p = 0.192) (Table 1).

During the baseline survey no health worker was trained on the new case-management policy. The follow-up survey results showed coverage of 21.5% of trained health workers (Table 1). With respect to the supervision, there was an increase from 41.5% to 51.9% (p = 0.026) of supervised health workers; however, the coverage of health workers who had received a supervisory visit that included any malaria case-management activity was low and without significant change between two survey rounds (17.9% vs 13.9%; p = 0.156). Similarly, there were no changes in the coverage of health workers who received supervisory visit that included observation of consultations (6.7% vs 6.8%; p = 0.981).

### Malaria diagnostic and treatment practices – policy performance and health workers adherence

Table 2 shows survey levels and changes in the performance of the composite case-management indicator and its components at all health facilities and at facilities with available AL and malaria diagnostics. At all facilities composite performance, defined as patient tested for malaria and treated with AL if the test result was positive or not treated for malaria if the test result was negative, was low during both survey rounds. Yet, there was a significant improvement from 15.7% at the baseline to 22.1% at the follow-up survey (p = 0.048). A similar upward trend was observed in testing rates – from 23.9% to 30.9% (p = 0.090). At facilities with available AL and malaria diagnostics, the performance of the same indicators was higher with a similar trend between surveys: composite performance increased from 28.1% to 35.5% (p = 0.114) and testing rates increased from 42.5% to 49.5% (p = 0.196).

**Table 2:** Levels and changes in key diagnostic and treatment indicators - performance of the new case-management policy (analysis at all health facilities) and health workers adherence to guidelines (analysis at facilities with available diagnostics and AL).

	Baseline survey		Follow-up survey		*P*-value
	n (%)	95% CI	n (%)	95% CI	
Policy performance	N = 2,405		N = 1,456		
Correctly managed^a^	378 (15.7)	12.0–19.4	321 (22.1)	16.7–27.3	0.048
Malaria test performed	575 (23.9)	18.9–28.9	450 (30.9)	24.4–37.4	0.090
Test positive treated with AL^b^	244 (82.7)	75.8–89.6	189 (89.2)	84.2–94.1	0.127
Test negative not treated with AM^c^	97 (34.6)	25.2–44.1	95 (39.9)	30.5–49.3	0.260

Health workers adherence	N = 1,239		N = 861		
Correctly managed^a^	348 (28.1)	22.6–33.6	306 (35.5)	27.8–43.3	0.114
Malaria test performed	526 (42.5)	35.9–49.0	426 (49.5)	40.9–58.0	0.196

Treatment for test positive	N = 276		N = 201		
AL	230 (83.3)	76.1–90.5	180 (89.6)	84.5–94.6	0.150
AL+QN	29 (10.5)	4.8–16.2	2 (1.0)	0–2.4	0.002
Other antimalarial^d^	15 (5.4)	1.6–9.2	14 (7.0)	2.3–11.6	0.607
No antimalarial prescribed	2 (0.7)	0–1.8	5 (2.5)	0.2–4.8	0.153
Antibiotic prescribed	176 (63.8)	55.3–72.2	123 (61.2)	50.8–71.6	0.699

Treatment for test negative	N = 250		N = 225		
AL	89 (35.6)	25.6–45.6	91 (40.4)	30.8–50.1	0.489
SP	27 (10.8)	2.6–19.0	6 (2.7)	0.1–5.2	0.022
Other antimalarial^e^	16 (6.4)	2.3–10.5	2 (0.9)	0–2.1	0.009
No antimalarial prescribed	118 (47.2)	37.0–57.4	126 (56.0)	45.7–66.3	0.227
Antibiotic prescribed	212 (84.8)	79.0–90.6	175 (77.8)	72.9–82.7	0.082

Treatment when test not done	N = 713		N = 435		
AL	394 (55.3)	46.7–63.8	184 (42.3)	32.8–51.8	0.046
Other antimalarial^f^	60 (8.4)	4.3–12.5	15 (3.5)	0.1–6.8	0.095
No antimalarial prescribed	259 (36.3)	27.8–44.8	236 (54.3)	43.1–65.4	0.013
Antibiotic prescribed	527 (73.9)	68.4–79.4	359 (82.5)	77.4–87.7	0.026

AL = artemether-lumefantrine; AM = antimalarial; QN = quinine; SP = sulfadoxine-pyrimethamine; AS = artesunate; AQ = amodiaquine; DHA = dihydroartemisinin; ART = artemether.

a Defined as management of febrile patient meeting all of the following three criteria: 1) patient tested for malaria; 2) if positive test result treated with AL, and 3) if negative test not treated for malaria.

b Denominators in this category include 295 patients at baseline and 212 at follow-up surveys.

c Denominators in this category include 280 patients at baseline and 238 at follow-up surveys.

d Other antimalarial treatment include QN (18), SP (3), AS+AQ (2), DHA (2), QN+SP (2), ART (1) and AL+SP (1).

e Other antimalarial treatment include AL+QN (8), QN (6), AQ (3) and AL+SP (1).

f Other antimalarial treatment include AL+QN (28), SP (28), QN (14), AQ (4) and DHA (1).

Beside low testing rates, case-management was further compromised with low health workers adherence to test negative results which despite some improvement trends (47.2% vs 56.0%; p = 0.227) was suboptimal. The suggested improvements were mainly due to a significant decline in the use of SP (10.8% vs 2.7%; p = 0.022) and other than AL antimalarial treatments (6.4% vs 0.9%; p = 0.009) (Table 2). Conversely, treatment practices of test positive patients with recommended AL were high at the baseline and have even shown some further improvement trends (83.3% vs 89.6%; p = 0.150). Among these patients, the practice of combining AL and quinine (AL+QN) which was present during the baseline (10.7%) became nearly non-existent during the follow-up survey (1.0%; p = 0.002). Finally, among febrile patients who were not tested for malaria, and therefore inappropriately managed according to new guidelines, there was a significant decline in the use of antimalarial drugs (63.7% vs 45.7%; p = 0.013), specifically AL (55.3% vs 42.3%; p = 0.046) yet an increased use of antibiotics (73.9% vs 82.5%; p = 0.026).

### Factors influencing malaria testing of febrile patients

Fifteen factors that may have influenced health workers decision to test for malaria at facilities where diagnostics were available are examined. Table 3 presents the multivariate model between factors and the outcome, and univariate results for factors of programmatic interest which did not meet the criteria for multivariate analysis (*P*-value<0.15). The multivariate results revealed significantly higher likelihood of testing practices at faith-based or non-governmental facilities compared to government facilities (OR = 2.43; 95% CI = 1.31–4.49), at facilities with malaria microscopy compared to those with RDTs (OR = 5.95; 95% CI = 1.90–18.65), at facilities with the caseload lower than 25 patients on survey day (OR = 1.99; 95% CI = 1.07–3.73), among patients 5 years and older (OR = 1.60; 95%: 1.05–2.45), and among febrile patients presenting without cough (OR = 1.51; 95% CI = 1.11–2.04), running nose (OR = 2.10; 95% CI = 1.32–3.33) and skin problem (OR = 2.55; 95% CI = 1.27–5.14). No significant association was found between the testing and exposure to the interventions such as in-service training on the new case-management policy (OR = 0.99; 95% CI = 0.48–2.05), supervisory visit including malaria case-management (OR = 1.11; 95% CI = 0.48–2.61) and access to malaria guidelines (OR = 0.53; 95% CI = 0.14–2.04) (Table 3).

**Table 3:** Predictors of health workers decision to test febrile patients for malaria.

Predictors	No of consultations	No (%)tested	OR(95% CI)	*P*-value
**Multivariate results**				
**Ownership of facility**				
Faith based or non-governmental	98	78 (79.6)	2.43 (1.31–4.49)	0.005
Government	763	348 (45.6)	1.0 (Ref.)	
**Type of malaria diagnostics at facility**				
Microscopy	822	417 (50.7)	5.95 (1.90–18.65)	0.002
Rapid diagnostic test	39	9 (23.1)	1.0 (Ref.)	
**Caseload on survey day**				
≤25 patients	355	216 (60.9)	1.99 (1.07–3.73)	0.031
>25 patients	506	210 (41.5)	1.0 (Ref.)	
**Age of patient**				
5 years and older	441	263 (59.6)	1.60 (1.05–2.45)	0.031
Below 5 years	420	163 (38.8)	1.0 (Ref.)	
**Cough main complaint**				
Absent	493	278 (56.4)	1.51 (1.11–2.04)	0.008
Present	368	148 (40.2)	1.0 (Ref.)	
**Running nose main complaint**				
Absent	778	401 (51.5)	2.10 (1.32–3.33)	0.002
Present	83	25 (30.1)	1.0 (Ref.)	
**Skin problem main complaint**				
Absent	817	413 (50.6)	2.55 (1.27–5.14)	0.009
Present	44	13 (29.6)	1.0 (Ref.)	
**Univariate results** a				
**HW trained on new case-management policy**				
Yes	211	104 (49.3)	0.99 (0.48–2.05)	0.979
No	650	322 (49.5)	1.0 (Ref.)	
**HW had supervisory visit including malaria**				
Yes	188	97 (51.6)	1.11 (0.48–2.61)	0.803
No	673	329 (48.9)	1.0 (Ref.)	
**HW has access to guidelines**				
Yes	29	10 (34.5)	0.53 (0.14–2.04)	0.353
No	832	416 (50.0)	1.0 (Ref.)	

HW = health worker.

a Only selected variables of programmatic importance are presented.

## Discussion

Universal coverage with health systems support activities and subsequent translation of these activities into universal adherence to the new case-management recommendations is an optimistic target to be achieved by 2013 at the health facility level in Kenya [7]. Our findings during 2010 identify several trends and gaps in the case-management which are directly relevant for the strengthening of the future implementation activities in Kenya.

### Coverage with health systems support activities

Absence of stock-outs of antimalarial drugs and malaria diagnostic services is a basic prerequisite for effective implementation of the new malaria case-management policy. Despite a declining, though statistically non-significant, trend in AL stock-outs (from 27% to 21% for all four AL packs and from 60% to 52% for at least one AL pack) the stock-out levels during 2010 were still substantial. This is of particular concern for simultaneous absence of all four AL packs which precludes effective treatment and is likely to be associated with increased childhood mortality as shown in Western Kenya [11]. Yet, ACT stock-outs revealed in our study are not unique reports – they had been reported from Uganda [12], Zambia [13], Nigeria [14], Sudan [15], Tanzania [16], Senegal [17], and indeed in the previous smaller studies in Kenya [10]. Similarly to AL availability, no significant changes were observed during 2010 in the capacity of health facilities to provide parasitological diagnosis resulting in an overall gap of 42% of facilities unable to provide either malaria microscopy or RDT diagnostic services. The findings on malaria diagnostic capacities in Kenya are not surprising given that these services are largely dependent on microscopy which is predominantly available at higher level facilities; however what is worrisome is that over two-thirds of facilities lacked RDTs in areas where RDTs had been supplied since 2006.

The investigations of the causes of commodity stock-outs are beyond the scope of this study and they deserve qualitative and quantitative examinations of the complete supply chain to comprehensively address these problems and ensure effective distribution systems. Yet, while further studies, programmatic strengthening of the supply chain including redistribution of stocks at peripheral level, and piloting of innovative approaches to eliminate ACT stock-outs such as recently demonstrated in Tanzania [18] should remain a priority, we also emphasize that rational use of antimalarial drugs based on malaria diagnostics must be viewed as an integral component of this process.

A minimum package of health workers' support activities necessary to implement, reinforce and maintain health workers' practices according to the case-management standards include provision of in-service training, guidelines and effective supervisory visits. Our findings revealed low coverage of these activities by the end of 2010; however, this was not a surprising finding and not in discordance with reports from several large scale evaluations at various stages of implementation process in other African countries [13], [14]. First, despite a national character of the training, 22% of trained health workers after the completion of the training programme correspond to the training capacities to cover 4,807 health workers within the universe of approximately 20,000 front-line health workers countrywide. Second, low access to the new national guidelines by the end of 2010 is due to lack of harmonization between the training implementation and guideline dissemination where the training, though based on new guidelines recommendations, took place prior to the printing and distribution of the guidelines. However, it was worrisome to observe that despite the majority of health workers receiving supervision, any malaria case-management activity was rarely a component of this activity. This is especially unfortunate given that the training was a nationwide and the routine supervision could have been a channel to reinforce translation of training messages into the clinical practice.

### Malaria case-management practices

We hypothesized that 15 percentage points is a minimum case-management improvement we would like to observe between two survey rounds to be able to substantially reduce the gap by 2013. Our findings revealed that during 2010 the policy performance increased from 16% to 22% of febrile patients managed according to the new guideline. While low performance rate at all study facilities can be explained by the absence of diagnostics and AL in nearly half of the facilities, at the facilities with available diagnostic services and AL, the performance of the same indicator, despite an increase of 8% between two survey rounds, remained however low (36%).

There are three levels of non-adherent health workers' practices resulting in poor performance of the composite indicator at facilities where commodities are available. First, the major discordance is related to low testing rates contributing currently to 78% of non-adherent practices. An increase in testing rates for febrile patients from 43% to 50% could be seen as an improvement trend, and indeed testing is higher than observed in larger scale evaluations in other countries [19]–[22] yet it is substantially lower than what was shown that can be achieved in Senegal [16] or under the smaller scale operational conditions in other countries [23], [24]. Second, despite a declining, yet statistically non-significant trend from 56% to 47% of patients with negative test results who are treated with an antimalarial, nearly half of the patients in this category are still treated in discordance with national guidelines. Recently, studies have shown that intensive interventions, including high quality of in-service training supported with supervision and strengthened monitoring and surveillance, can substantially improve adherence to test negative results [23]–[27]. However, as reported from a number of countries, and concurring with our findings, the adherence challenges remain under both, microscopy and RDT diagnostic strategies, when routine programmatic interventions are evaluated on larger scale and in less controlled conditions [20]–[22], [28]–[31]. Several studies have also reported that an important component facilitating health workers' adherence to test negative results is development and implementation of guidelines for management of non-malaria febrile illness [23], [32]. Despite a standardization of guidelines and manuals during 2010 this is a still pending component of the case-management activities in Kenya that should be addressed as part of collaborative efforts between different national programmes. Third, we are glad to report that six years following the change of treatment policy, the lowest discordance was found in patients group with positive test result where use of recommended AL treatment reached 90% at the end of 2010 and non-adherent treatment practices observed in prior years [33], [34], and up to certain extent during the baseline survey, became very rare.

### Predictors of health workers decision to test patients for malaria

Beside the absence of malaria testing services, non-adherence to testing recommendations where diagnostics are available present a major impediment compromising performance of the new case-management-policy. Our predictors analysis brings an additional light on factors influencing health workers' testing practices. Importantly, we observed that exposure to in-service training on the new recommendations, supervision and guidelines have not influenced testing practices. The in-service training for health workers, as the main case-management implementation activity during 2010, deserves attention. The deficiencies and limited effectiveness of stand-alone in-service trainings were previously reported in Kenya [35], [36] and in other parts of Africa [12], [29]. In 2010 the suboptimal training's effect could be attributed to the uncertain quality of the training implementation, absence of post-training follow-up component, qualitative and quantitative deficiencies of supportive supervision at health facilities to reinforce practices, and implementation of the training prior to the distribution of the new guidelines.

Yet, several other factors influenced health workers decision to test for malaria. First, patients were twice more likely to be tested at facilities with lower caseload, the finding also observed recently in Angola [22]. Second, patients were six times more likely to be tested at facilities with malaria microscopy compared to those providing RDTs - the finding suggesting health workers preference for microscopy or possible lack of trust in RDT based malaria diagnosis. Third, testing was more common for patients above 5 years of age, what is likely a reflection of long term policy promoting presumptive treatment in young children. Fourth, patients at faith based facilities were more likely to be tested than in government owned, what may be due to more established cost-recovery schemes at these facilities with higher testing charges attracting economically wealthier patients. Finally, patients presenting with fever but without complaints of cough, running nose and skin problem were also more likely to be tested. The findings may suggest health workers' intention to rule out malaria in febrile patients on clinical grounds, however the practice deemed inappropriate since it is inconsistent with Kenyan guidelines, the prior research showing lack of clinical algorithms to reliably rule out malaria [37]–[39], and finally with this study context where 39–44% of febrile patients who were tested and presented with cough, running nose and skin problem were also positive for malaria (data available upon request).

### Conclusion

The findings at the end of the first year of the implementation process, and two years before midterm evaluation of the 2009–2017 NMS, suggest that most of the key indicators have shown some improvement trends, however the differences observed were smaller than expected, rarely statistically significant, and resulting for the majority of the indicators in a substantial coverage and performance gap to be bridged in the next two years. To reduce case-management gaps towards 2013 targets, the opportunity lies in the forthcoming scale-up of RDTs, however the success of the activity will be critically dependent upon the delivery of a comprehensive case-management package. The minimum content of this package should include high quality of the training focusing on the deficiencies highlighted in this study, alignment of the training with RDT distribution, and importantly translation of stand-alone activities into post-training follow-up, improved quantity and quality of the supervisory visits, and more intense routine monitoring at district level able to overcome inherited barriers and weaknesses of the health systems. Failure to deliver comprehensive package of case-management interventions would risk leaving an important gap towards the optimistic targets.
